# Non-targeted metabolite profiling reveals changes in oxidative stress, tryptophan and lipid metabolisms in fearful dogs

**DOI:** 10.1186/s12993-016-0091-2

**Published:** 2016-02-12

**Authors:** Jenni Puurunen, Katriina Tiira, Marko Lehtonen, Kati Hanhineva, Hannes Lohi

**Affiliations:** Institute of Public Health and Clinical Nutrition, University of Eastern Finland, Kuopio, Finland; Department of Veterinary Biosciences and Research Programs Unit, Molecular Neurology, University of Helsinki, Biomedicum Helsinki, P.O. Box 63, 00014 Helsinki, Finland; The Folkhälsan Research Center, Helsinki, Finland; School of Pharmacy, University of Eastern Finland, Kuopio, Finland; LC–MS Metabolomics Center, Biocenter Kuopio, Kuopio, Finland

**Keywords:** Dog, Anxiety, Fear, Non-targeted metabolite profiling, Metabolomics

## Abstract

**Background:**

Anxieties, such as shyness, noise phobia and separation anxiety, are common but poorly understood behavioural problems in domestic dogs, *Canis familiaris*. Although studies have demonstrated genetic and environmental contributions to anxiety pathogenesis, better understanding of the molecular underpinnings is needed to improve diagnostics, management and treatment plans. As a part of our ongoing canine anxiety genetics efforts, this study aimed to pilot a metabolomics approach in fearful and non-fearful dogs to identify candidate biomarkers for more objective phenotyping purposes and to refer to potential underlying biological problem.

**Methods:**

We collected whole blood samples from 10 fearful and 10 non-fearful Great Danes and performed a liquid chromatography combined with mass spectrometry (LC–MS)-based non-targeted metabolite profiling.

**Results:**

Non-targeted metabolomics analysis detected six 932 metabolite entities in four analytical modes [RP and HILIC; ESI(−) and ESI(+)], of which 239 differed statistically between the test groups. We identified changes in 13 metabolites (fold change ranging from 1.28 to 2.85) between fearful and non-fearful dogs, including hypoxanthine, indoxylsulfate and several phospholipids. These molecules are involved in oxidative stress, tryptophan and lipid metabolisms.

**Conclusions:**

We identified significant alterations in the metabolism of fearful dogs, and some of these changes appear relevant to anxiety also in other species. This pilot study demonstrates the feasibility of the non-targeted metabolomics and warrants a larger replication study to confirm the role of the identified biomarkers and pathways in canine anxiety.

## Background

Anxiety-related disorders, including compulsions, fearfulness, noise phobia, generalized anxiety and separation anxiety, are common but complex and poorly understood behavioural problems in domestic dogs (*Canis familiaris*) [1–3]. Clinical, ethological and pharmacological studies suggest that the underlying biochemical mechanisms are shared in dogs and humans. This is demonstrated, for example, by a successful treatment of the dogs with human anxiolytes [4]. Given the biological similarity of canine and human anxiety, dogs with a particular genomic system could serve as a feasible gene discovery model for human anxiety and improve the molecular understanding of the disease in general. Breed-specificity of many anxieties, such as canine compulsive disorder, suggests genetic susceptibility [4–6]. However, environmental factors, such as negative experiences and poor socialization during puppyhood, affect also behavior [7–9] and complicate gene discoveries, which are still rare [10–14].

One of the challenges in anxiety research concerns objective behavioural measurement to establish valid research cohorts for gene discovery. Current approaches rely on behavioural questionnaires and tests, which appear to correlate well [2] but have intrinsic limitations related to subjectivity and temporality, respectively. There is a need for more objective measures such as physiological biomarkers, which could help not only phenotyping but could also refer to the underlying affected molecular pathways. High-throughput –omics technologies such as metabolomics could facilitate discovery of biomarkers for research, diagnostics and treatment options. Non-targeted metabolite profiling offering a hypothesis-free approach can detect molecular biosignatures and has been successfully applied to identify genetic and environmental contributions to diseases [15–17]. For example, metabolic profiling of schizophrenia has revealed changes in glutamine and arginine metabolism, which may reflect genetic susceptibility to this neuropsychiatric disorder [18].

In this pilot study, we aimed to compare metabolite profiles of fearful and non-fearful dogs to identify fear-related pathways and biomarkers for more objective phenotyping. We have previously developed a validated approach for anxiety phenotyping in dogs [2] to select 10 fearful and 10 non-fearful Great Danes. We analysed whole blood samples using a non-targeted LC-qTOF-MS metabolomics method to compare the metabolic profiles. Our results reveal changes in several anxiety-relevant components in fearful dogs and warrant a larger metabolomics study in canine anxiety to replicate the findings in this pilot study.

## Methods

### Animals and study design

The dogs were selected from our previously established anxiety research cohort [2], which included a validated owner-filled anxiety questionnaire and a behavioural test for part of the dogs (4 out of 10 controls and 3 out of 10 cases). The questionnaire survey included both general questions concerning dog’s behavior in various situations (such as meeting unfamiliar people, dogs, and behavior in new situations, and when exposed to loud sounds) and daily routines, and also several more specific background questions concerning the early experiences of the dog, related to e.g. puppy period and socialization [2]. Based on the data from the questionnaire, several behavioral variables were derived and used to select dogs to the study groups. The variables that we were interested the most were fear towards unfamiliar people (human fear_frequency, human fear_intensity), fearfulness total and noise sensitivity. Human fear_frequency was simply the owner reported frequency of dog showing fearful reaction when meeting a stranger (frequency scoring 0 = never; 1 = 0–40 % of the occasions; 2 = 40–60 % of the occasions; 3 = 60–100 % of the occasions: 4 = always when meeting unfamiliar people). Human fear_intensity was calculated as follows: the frequency of showing fearful reaction when meeting unfamiliar people was multiplied with the sum of owner recorded fearful behavioral reactions. Each type of behavior equaled 1, except the avoidance-reaction which was weighted by multiplying it with 5. Fearfulness variable was calculated as a sum of frequencies of showing fearful behavioural reactions towards unfamiliar people (see scoring above 0–4), unfamiliar dogs (0–4) and in new situations (0–4), and thus the score varied between 0 and 12. In addition, we calculated a variable describing the dog’s fear of loud noises (noise sensitivity), by calculating a sum of frequencies of showing a fearful reaction towards thunder (see scoring above 0–4), fireworks (0–4) and gunshot (0–4). The behaviour of seven of the dogs was verified by a short 5-min test conducted by same person for all the dogs—not all dogs were tested as some had already died between the blood sampling and behavioral testing, or lived too far. Shortly, test consisted of three parts; meeting an unfamiliar person, exploration in the novel space, and novel object test. More details of the test can be found from Tiira and Lohi, 2014.

We selected 10 fearful and 10 non-fearful Great Danes for the study, and detailed information about all the individual dogs is presented in Table 1. Our criteria for non-fearful dogs was that all the variables (human fear_frequency, human fear_intensity, fearfulness total and noise sensitivity) had to have score 0. In the case group, our main inclusion criterion was that the dog had to show fear towards unfamiliar people at 40–100 % of all situations (human fear_frequency score 2–4). In addition, dog’s needed to have fearfulness score >2. Additionally to these criteria, we used matched pairs with approximately same age for blood samples between case and control groups. We aimed, at first, to get only males, however, in order to keep the age of blood sampling approximately same in both control and case groups we also had to include two females for both groups. EDTA-blood samples were collected from each dog and stored in −20 degrees. The blood samples were collected from the privately owned Finnish dogs with owners consent under a valid ethical license (Finnish National Animal Experiment Board, ELLA, license number ESAVI/6054/04.10.03/2012).

**Table 1:** Demographics of the dogs

	Age (years)	Mean age (SD)	Sex	Fearfulness (total)	Human fear_frequency	Human fear_intensity	Noise sensitivity	Behavioral test	Diet
1	1.1	3.5 (2.5)	Male	4	3	10	3	No	Not known
2	1.5		Male	10	2	16	2	No	Dry food, raw meat, oils
3	2.4		Male	3	4	21	3	No	Dry food
4	2.8		Male	7	4	28	0	No	Raw food, oils
5	4.2		Male	10	3	30	0	Yes	Not known
6	5.4		Male	6	2	14	5	No	Dry food
7	4.4		Male	8	2	14	4	Yes	Not known
8	9.3		Male	6	3	6	8	No	Dry food
9	1.8		Female	8	4	28	0	No	Dry food, meat, fish
10	1.6		Female	10	4	30	0	Yes	Raw food, oils
11	3.2	3.4 (2.2)	Male	0	0	0	0	No	Raw food, oils
12	4.5		Male	0	0	0	0	No	Dry food
13	3.3		Male	0	0	0	0	Yes	Dry food, oils
14	1.1		Male	0	0	0	0	No	Not known
15	8.5		Male	0	0	0	0	Yes	Dry food
16	4.8		Male	0	0	0	0	Yes	Dry food, meat
17	3.3		Male	0	0	0	0	No	Dry food, oils
18	1.6		Male	0	0	0	0	No	Dry food, oils, vitamin C
19	2		Female	0	0	0	0	Yes	Homemade food, meat, dry food, oils
20	2.1		Female	0	0	0	0	No	Not known

Detailed information, including age, sex, behavioral scores and diet, is provided for each individual dog. Dogs numbered from 1 to 10 are fearful dogs, whereas dogs numbered from 11 to 20 are non-fearful dogs

### Dietary information

The owners were retrospectively asked to report the diet of the dog at the time of blood sampling to help us consider possible nutritional effects on metabolite profiles. Dietary information was collected from 17 out of 20 dogs (two cases and one control missing). Comparison of the diet profiles indicated only minor differences between the test groups. The diets contained equally a mix of raw food, commercial dry foods, homemade food and different dietary supplements in both test groups. However, the dietary profiles varied greatly within the test groups but similar variations were observed in both groups. The basic contents of all commercial dry foods fed to the dogs were rice, chicken meal, pork meal, maize, fish oil, animal fat, vegetable fibre, and beet pulp in addition to minerals, such as calcium (Ca) and phophorus (P), micronutrients, such as iron (Fe), copper (Cu), zinc (Zn) and iodine (I), and vitamins, such as vitamins A, D_3_ and E. Interestingly, there were minor differences in the intake of pulses between case and control dogs, since the commercial dry foods eaten by a few control dogs but not cases contained soybean oil, soybean meal and pea bran meal.

### Non-targeted LC–MS metabolite profiling analysis

The non-targeted LC-qTOF-MS-analysis and preprocessing of raw data were performed in the LC–MS Metabolomics Center at Biocenter Kuopio (University of Eastern Finland). For metabolite extraction, 400 µL of acetonitrile was added to 100 µL of whole blood sample, and mixed in vortex at maximum speed 15 s. The samples were incubated on ice bath for 15 min, and centrifuged at 16000×*g* for 10 min in order to collect the supernatant. The supernatants were filtered into HPLC vials using 0.2 μm Acrodisc^®^ Syringe Filters with a PTFE membrane (PALL Corporation, Ann Arbor, MI) prior subjecting to the LC–MS analyses. From every extracted sample, aliquots of 10 µL was taken and combined in one tube, and used as the quality control (QC) sample in the analysis.

The whole blood samples were analysed by the UHPLC-qTOF-MS system (Agilent Technologies, Waldbronn, Karlsruhe, Germany) that consisted of a 1290 LC system, a Jetstream electrospray ionization (ESI) source, and a 6540 UHD accurate-mass qTOF spectrometer. The samples were analyzed using two different chromatographic techniques, i.e. reversed phase (RP) and hydrophilic interaction chromatography (HILIC) to maximize metabolome coverage. The RP chromatography was performed on Zorbax Eclipse XDB-C18 column (100 × 2.1 mm, 1.8 µm, Agilent Technologies, Palo Alto, CA, USA). The temperature of the column was kept on 50 °C, and the flow rates of mobile phases were set as 0.4 mL/min. The mobile phases consisted of water (eluent A) and methanol (eluent B), both containing 0.01 % (v/v) of formic acid. The gradient profile employed was as follows: 2 → 100 % B (0–10 min); 100 % B (10–14.5 min); 100 → 2 % B (14.5–14.51 min); 2 % B (14.51–16.50 min). The injection volume in RP was 2 µl. The HILIC chromatography was performed on Acquity UPLC BEH Amide column (100 × 2.1 mm, 1.7 µm; Waters Corporation, Milford, MA), and the temperature of the column was kept on 45 °C. The flow rate was 0.6 mL/min, and eluents A and B consisted of 50 % v/v and 90 % v/v ACN, respectively, both containing 20 mM ammonium formate. The gradient was as follows: 100 % B (0–2.5 min); 100 → 0 % B (2.5–10 min); 0 → 100 % B (10–10.01 min); 100 % B (10.01–12.5 min). The injection volume in HILIC was 2 µl.

The MS ion source conditions were as follows: ESI source, operated both in positive (+ve) and negative (−ve) ionization mode, drying gas temperature 325 °C with a flow of 10 L/min, sheath gas temperature 350 °C and flow 11 L/min, nebulizer pressure 45 psi, capillary voltage 3500 V, nozzle voltage 1000 V, fragmentor voltage 100 V, and skimmer 45 V. For data acquisition, the mass range was 20–1600 amu with acquisition rate 1.67 spectra/s. In order to get the automatic MS/MS spectrums, four ions with the highest intensities were selected from every precursor scan cycle for fragmentation performed on the QC samples. After two product ion spectra, these ions were excluded, and released again for fragmentation after a 0.25-min hold. The collision energies were 10, 20 and 40 V. If the molecular ion of a compound was not included into automatic MS/MS fragmentation, targeted MS/MS analyses with collision energies 10 and 20 V were conducted. A continuous mass axis calibration was performed by monitoring two reference ions from an infusion solution throughout the runs. In positive mode the reference ions were *m/z* 121.050873 and *m/z* 922.009798, and in negative mode *m/z* 112.985587 and *m/z* 966.000725. Data acquisition was conducted with MassHunter Acquisition B.04.00 (Agilent Technologies). The QC samples were injected in the beginning and ending of the analysis and also after every 10 samples.

### Non-targeted metabolomics data analysis

#### Data collection and statistical analysis

The LC–MS data was collected using the vendor’s software MassHunter Qualitative Analysis B.05.00 (Agilent Technologies), where the ions were extracted to compounds utilizing the “Find by molecular feature” algorithm. The data were output as compound exchange format (.cef-files) into the Mass Profiler Professional software (MPP 2.2, Agilent Technologies) for compound alignment, data preprocessing, and statistical analysis (Student’s t test between the case and control groups). In order to reduce noise and remove insignificant metabolite features, only the features found in at least 60 % of the samples in at least one replicate group (case or control) were included in the analysis. This resulted in a dataset comprising 6 932 features in four separate analytical runs [986 in HILIC ESI(+), 1 071 in HILIC ESI(−), 3 790 in RP ESI(+), and 1 085 in RP ESI(−)].

The pre-processed data from each of the four analytical approaches were subjected to supervised classification algorithm partial least-squares discriminant analysis (PLS-DA; Simca-13, Umetrics, Sweden). The data were log10-transformed, pareto-scaled and the model was validated by the Simca-13 internal cross validation, and the resulting variable importance projection (VIP) values for each metabolite [19, 20], were integrated in the data. The PLS-DA illustrates the differences between case and control groups by investigating those metabolites that are the largest discriminators in the data, and the larger the VIP value is, the more significant contributor the metabolite is in the model.

The data was filtered according to VIP >1 in order to reduce insignificant features from the data, resulting in a dataset comprising 2 114 features in the four analytical runs [308 in HILIC ESI(+), 301 in HILIC ESI(−), 1 162 in RP ESI(+), and 343 in RP ESI(−)]. After adjusting for multiple comparisons by Benjamini-Hochberg false discovery rate (FDR) correction [21] (R project for Statistical Computing version 3.0.1.) within each of the four analytical approaches, the peak lists were filtered according to uncorrected p value <0.05, fold change (FC) ≥±1.2, PLS-DA VIP >1, and feature present in at least seven replicates in either of the groups. This resulted in dataset of 239 entities [45 in HILIC ESI(+), 41 in HILIC ESI(−), 127 in RP ESI(+), and 26 in RP ESI(−)], where the compounds having FDR corrected p value <0.05 were considered as statistically significant differences between control and case groups, whereas those with uncorrected p value <0.05 were regarded nominally significant. In addition, the filtered data were subjected to the K-means cluster algorithm with the Pearson correlation as distance metric followed by the hierarchical cluster analysis and heat-map output for data visualization [22].

Finally, the remaining peaks in the lists were manually inspected in the LC–MS chromatograms and spectra with the MassHunter software to locate peaks with poor retention and peak shape, which were filtered out from further analysis. Peak lists were also looked through to ensure that the molecular ion of a compound was included into data dependent MS/MS analysis, and in case not, targeted MS/MS analysis was performed.

#### Identification of the differential features in the LC–MS data

The identification of metabolites was based on the accurate mass and MS/MS fragmentation spectra acquired either in the automatic, data dependent MS/MS analysis during the initial data acquisition, or via re-injection of the samples in targeted MS/MS mode. The spectra were compared against The METLIN Metabolite Database (https://metlin.scripps.edu/index.php), Human Metabolome Database (HMDB) (http://www.hmdb.ca/), and LipidMaps (http://www.lipidmaps.org/), or fragmentation patterns reported in earlier publications. The identification of lipids was based on their characteristic fragmentation patterns reported in earlier publications [23–25]. The key elements for identification were the protonated head group (*m/z* 184.07 for PCs and LysoPCs, and *m/z* 196.03 for PEs) as well as the deprotonated fatty acid fragments visible in the negative ionization mode (the MS/MS fragmentation data for all of the identified metabolites is presented in Table 1). The identification of plasmalogen was based on the *m/z* 303 corresponding to arachidonic acid (C20:4), and on the characteristic fragmentation pattern of phosphoethanolamine plasmalogens (PEP) described previously [26].

## Results

A non-targeted LC–MS-based metabolomics platform was used to compare the whole blood metabolite profiles of fearful and non-fearful dogs. The two test groups had similar overall dietary profiles with a note that many control dogs were reported to consume more protein-rich food such as soybeans than cases. We detected a total of 6 932 molecular features in the four separate LC–MS runs, of which 239 were differential between the two groups (Student’s t-test, p value < 0.05; FC ≥±1.2; PLS-DA VIP >1). This set of compounds (239) was subjected to manual inspection to identify metabolites and to remove redundant ions as well as poorly retained and integrated peaks. This analysis resulted in a set of 13 known metabolites and 5 unknown features (Table 2).

**Table 2:** Characteristics for the putatively identified marker metabolites in liquid chromatography-mass spectrometry analysis

	Column	Ionization mode	MW	*m/z*	RT (min)	Putative annotation	p-value^a^	FDR corrected p-value^b^	Fold change (FC)^c^	CID (eV)	MS/MS fragmentation	Identification reference^d^	VIP
Cluster 1	RP	ESI+	821.577	822.584	10.67	PC(16:0/23:5)	0.0017	0.0226	−2.06	20	822.584, 184.074; ESI(−) 40 eV: 806.556, 343.249, 255.233	MS/MS	2.24
	RP	ESI+	801.587	802.595	11.35	PC(18:0/19:1)	0.0376	0.1316	−1.98	20	184.072, 784.5833, 802.594; ESI(−) 40 eV: 295.229, 283.261, 786.565	MS/MS	1.30
	RP	ESI+	204.09	205.097	2.30	Tryptophan	4.22E−04	0.0087	−1.58	10	188.0698, 146.0599, 144.0806, 130.0613, 132.0788, 159. 0881, 205.0947	Standard	0.84
	RP	ESI+	537.295	538.309	10.25	LysoPC(19:0)	0.0488	0.1461	−1.53	20	104.106, 501.236, 560.310 [M + Na]^+^; ESI(−) 20 eV: 522.323, 297.245	MS/MS	1.68
Cluster 2	RP	ESI−	579.319	578.312	8.79	Unknown LysoPC	0.0103	0.0884	−2.79	20	293.209, 578.310, 518.291; ESI(+) 20 eV: 104.107, 534.319, 184.074	MS/MS	2.08
	HILIC	ESI−	88.016	87.009	1.77	Unknown metabolite	0.0427	0.1108	−2.58	10	44.999, 73.857	MS/MS	1.34
	RP	ESI+	809.592	810.599	12.64	PC(18:0/20:4)	0.0200	0.0965	−2.00	40	184.073, 86.095; ESI(−) 40 eV: 303.234, 283.265, 794.567	MS/MS	1.82
	RP	ESI+	517.316	518.323	8.81	LysoPC(18:3)	0.0472	0.1443	−1.86	40	184.072, 104.104, 86.094, 60.082; ESI(−) 20 eV: 502.2945, 277.2162	MS/MS	1.47
	RP	ESI+	499.270	500.277	9.17	LysoPE(20:5)	0.0416	0.1376	−1.64	10	500.2786, 359.2548; ESI(-) 20 eV: 498.2860, 169.1368, 301.2172	MS/MS	1.28
Cluster 3	RP	ESI+	311.319	312.326	11.01	Unknown metabolite	0.0240	0.1055	2.85	20	312.326, 57.071, 102.095, 100.075, 214.214, 81.068	MS/MS	1.88
	HILIC	ESI−	189.994	188.986	0.69	Pyrocatechol sulfate	0.0150	0.0734	2.36	40	108.024, 79.957, 53.042, 80.965, 109.027	Pyrocatechol standard	1.55
	HILIC	ESI+	246.137	247.144	1.42	Hypaphorine	0.0485	0.1719	2.17	10	188.071, 60.081, 146.061, 55.017, 247.206, 144.079, 85.0245, 118.928	Keller et al. [34]	1.29
	RP	ESI−	213.009	212.002	2.43	Indoxylsulfate	0.0480	0.1870	1.78	10	212.007, 80.966, 132.043	MID 253	1.85
	RP	ESI+	370.308	371.315	10.96	Unknown fatty acyl, either di-(2-ethylhexyl)adipate or dioctyl hexanedioate	0.0335	0.1253	1.55	10	129.0557, 111.0459, 147.0635, 101.0612, 57.0694, 241.1772	MS/MS	1.60
	RP	ESI+	315.277	316.285	8.7	Unknown sphingosine, either dehydrophytosphingosine, 6-hydroxysphingosine, or (4OH,8Z,t18:1) sphingosine	0.0087	0.0619	1.50	40	93.071, 43.055, 57.069, 81.070, 69,069, 67.055, 95.048, 77.040	MID 392	0.68
	RP	ESI+	283.287	284.294	10.59	Stearamide	0.0266	0.1108	1.28	20	284.295, 57.070, 102.091, 88.076, 71.085, 43.054	MID 34494	1.73
Cluster 4	HILIC	ESI+	136.039	137.046	1.43	Hypoxanthine	0.0250	0.1225	1.87	20	137.046, 119.035, 94.040, 110.035, 55.029, 82.038	MID 83	1.60
	RP	ESI−	749.54	748.531	12.51	PE(P-18:1/20:4)	0.0447	0.1866	1.81	20	748.526, 303.234; ESI(+) 20 eV: 361.275, 390.2773, 609.529, 750.551	MS/MS	1.61

Also the most significant non-identified marker metabolites are included. The characteristics include both uncorrected and FDR corrected p values, fold changes, Variable influence on projection (VIP) –values, and identification references, together with parameters for the LC–MS analysis, including the chromatography (Column), ionization mode in the mass spectrometry (Ionization mode), molecular weight (MW), identified ion (*m/z*), retention time (RT), collision induced dissociation energy (CID), and fragment ions in the tandem mass spectrometry (MS/MS fragments). n = 20 dogs (10 fearful and 10 non-fearful dogs). Note that two metabolic features, tryptophan and unknown sphingosine, are included in the table despite their low VIP values, since they otherwise show statistical significance

*LysoPC* lysophosphatidylcholine, *LysoPE* lysophosphatidylethanolamine, *PC* phosphatidylcholine, *PE* phosphatidylethanolamine

^a^Student’s t-test comparing the fold change against the Control group. P values <0.05 were considered as statistically significant

^b^Benjamini–Hochberg false discovery rate (FDR) corrected p value

^c^Average fold change when compared against the Control group, with p values. Fold changes ≥±1.2 were considered as statistically significant. Positive values indicate increased whole blood levels in case dogs vs. control dogs, whereas negative values indicate decreased whole blood levels in case dogs vs. control dogs

^d^Identification of metabolites is based on manual MS/MS spectral interpretation, METLIN ID when MS/MS spectrum available, commercial standard compound, or some earlier published fragmentation patterns. Keller et al. [34]

### Several phospholipids were differential between fearful and non-fearful dogs

Majority of the significantly changed metabolites in canine whole blood were identified as phospholipids, including phosphatidylcholines (PC), lysophosphatidylcholines (LysoPC), phosphatidylethanolamine plasmalogen (PEP) and lysophosphatidylethanolamine (LysoPE). Majority of them were decreased in the group of fearful dogs, especially PC(16:0/23:5) (−2.1-fold; Pcorr = 0.0226), PC(18:0/20:4) (−2.0-fold; P = 0.02) and PC(18:0/19:1) (−2.0-fold; P = 0.0376) showed remarkable differences between the two test groups. Additionally, an unknown lipid with m/z 578.312 (−2.8-fold; P = 0.0103), which exhibited similar fragmentation pattern to LysoPCs, was detected. Furthermore, a metabolite with *m/z* 748.531 was regarded as a nominally increased in fearful dogs (1.8-fold; P = 0.0447). The fragmentation suggested this compound to be PE(P-18:1/20:4), a phosphatidylethanolamine plasmalogen belonging to subclass of ether-linked lipids that are characterized by an ether linkage at the sn-1 position and an ester-linkage at the sn-2 position on the glycerol backbone of the lipid [26, 27].

### Oxidative stress and tryptophan pathways affected in fearful dogs

We found also several metabolites related to oxidative stress and tryptophan pathways that were changed between fearful and non-fearful dogs. Two compounds, *m/z* values of 137.046 (1.9-fold; P = 0.025) and 212.002 (1.8-fold; P = 0.048), showed identical fragmentations with hypoxanthine (MID 83) and indoxylsulfate (MID 253) in METLIN, respectively. Both of these metabolites are known to promote oxidative stress [28–32], and indoxylsulfate is an indole-derivated metabolite of tryptophan [32]. Metabolite with *m/z* 247.144 (2.2-fold; P = 0.0485) was identified as hypaphorine, a methylated form of tryptophan, based on its similar fragmentation pattern with the previously published spectra [33, 34]. We found also lower levels of tryptophan among fearful dogs (−1.6-fold, Pcorr = 0.0087), although the significance of this finding is questionable since tryptophan was detected in altered levels only in RP analysis and not in HILIC analysis. The latter would be more reliable method to detect amino acids.

### Other metabolic changes in fearful dogs

Another particularly clear change in the metabolite profiles of the two test groups was the accumulation of pyrocatechol sulfate, a phenolic metabolite with *m/z* 188.986 (2.4-fold; P = 0.015). It was identified based on fragmentation match with pyrocatechol standard compound, and additional fragment ion at *m/z* 79.957 corresponding to sulfate group [SO_3_]^−^ in the molecular structure of the compound. Additionally, a compound with *m/z* 284.294 and rt 10.59 in the RP ESI(+) analysis was observed to accumulate in case group (1.3-fold; P = 0.0266) and identified as stearamide (MID 34494), a fatty amide found in food packaging materials according to Human Metabolome Database (HMDB).

The most remarkable accumulation in case group was observed for a compound with *m/z* 312.326 and rt 11.01 in the RP ESI(+) analysis (2.8-fold; P = 0.024). However, this compound remained unidentified due to its unknown fragmentation pattern, although the retention time highly suggests fatty acid structure. The identity of three other metabolic markers remain also unclear, since compound with *m/z* 87.009 (−2.6; P = 0.0427) would match with pyruvate by its mass but its MS/MS fragmentation pattern was not identical with the spectrum in METLIN, whereas the feature with *m/z* 371.315 (1.6-fold; P = 0.0335) in the RP ESI(+) analysis showed similar fragmentation to two fatty acyls, di-(2-ethylhexyl)adipate and dioctyl hexanedioate, although could not be distinguished from each other. A metabolite with *m/z* 316.285 and rt 8.7 has MS/MS fragmentation similar to sphingosines, but due to the lack of published spectra, its exact identity remains unclear.

### Chemometric analysis of the LC–MS data

**Fig. 1:**
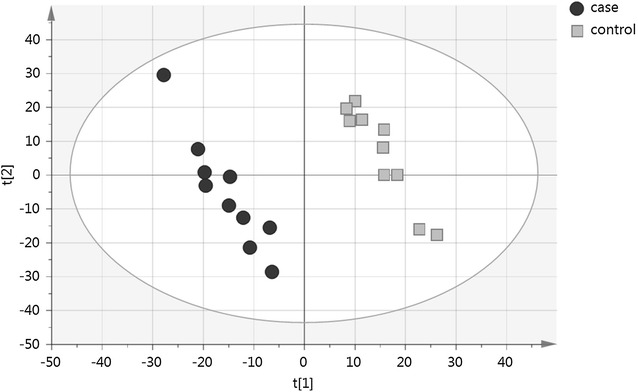
Partial least squares discriminant analysis (PLS-DA) of the reversed phase positive ESI–MS mode data. The *score plot* shows the individual samples in both case and control groups. Case group (*black circles*); Control group (*grey squares*)

The partial least squares discriminant analysis (PLS-DA) analysis yielding variable influence projection (VIP) values for metabolites indicated that the most important discriminator metabolites, i.e. those metabolites with high VIP values, had usually also low p-values and high fold changes, being prominent candidate biomarkers (e.g. PC(16:0/23:5): Pcorr = 0.0226, VIP = 2.24) (Table 2). Moreover, the PLS-DA analysis also clearly visualized the differences between control and case dogs, as exemplified with the data from the RP ESI(+) mode (Fig. 1).

**Fig. 2:**
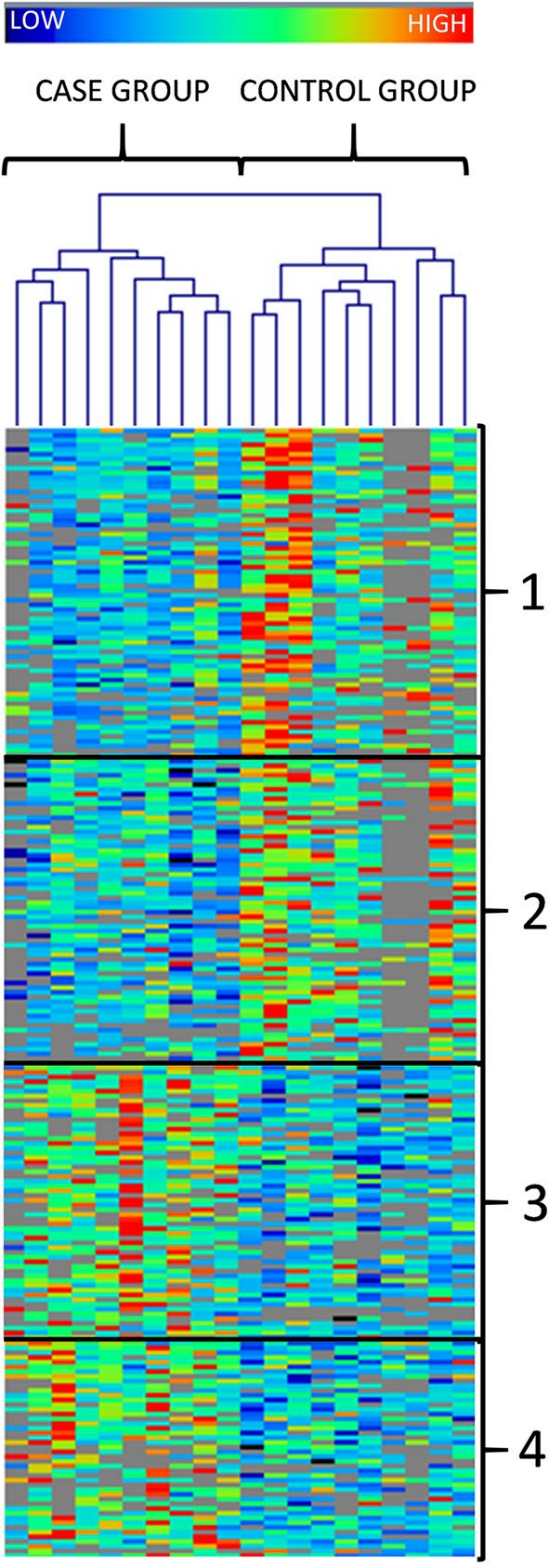
K-means cluster analysis with the hierarchical clustering of the fold change values (case versus control). Included are the metabolic features having p value <0.05 and fold change value ≥±1.2. The clusters are numbered one–four as depicted in the figure. The values for peak areas were row-wise normalized, and the *colour scale* indicates high (*red*) or (*low*) metabolite level. *Grey colour* in the heat map indicates not detected value

The 239 differential features were also subjected to the K-means cluster algorithm followed by hierarchical cluster analysis giving a heat map as an output (Fig. 2). Four clusters were formed. Cluster 1 contained a set of 70 decreased metabolites among case group, including identified PC(16:0/23:5), PC(18:0/19:1), LysoPC(19:0) and tryptophan. Also cluster 2 included features having lower concentrations in case group but with larger diversity among the samples. The third group clustered 58 compounds increased among fearful dogs, including pyrocatechol sulfate, hypaphorine, indoxylsulfate, stearamide, sphingosine-like molecule, putative fatty acyl, and one unknown feature with sharp and large peak. Cluster 4 indicated hypoxanthine and PE(P-18:1/20:4) together with several unknown metabolites having higher concentrations among fearful dogs. Hierarchical cluster analysis also revelead the relatively high degree of heterogeneity between the samples, especially within the control group (Fig. 2).

## Discussion

Anxiety-related disorders are common but yet poorly characterized for molecular underpinnings in any species. Research is challenged by clinical and genetic heterogeneity and there is a need for novel biomarkers to pinpoint affected pathways, to improve diagnostics, and to support research. This pilot study with non-targeted metabolomics addressed canine fear to establish methodology and to compare metabolic profiles in fearful and non-fearful dogs in order to elucidate the molecular phenomena related to anxiety. We identified 13 differential metabolites which indicated decreased phospholipids, elevated levels of the metabolites in oxidative stress pathways, and altered tryptophan metabolism in fearful dogs.

About half of the identified 13 metabolites were phospholipids, including three PCs, two LysoPCs, one LysoPE and one phosphatidylethanolamine plasmalogen. PCs, LysoPCs and LysoPE were all decreased and only plasmalogen elevated in fearful dogs. Phospholipids are major components of cell membranes and important signalling molecules [35]. Together with fatty acids they have been associated with anxiety-related diseases and behavior in humans and mice [17, 35–41]. In schizophrenia patients, for example, lower levels of plasma PEs and PCs have been measured when compared to healthy controls, suggesting an involvement of lipid disorder in schizophrenia [42]. Since the blood lipid composition is strongly affected by nutrition [43], the observed difference in the phospholipid levels could originate from diet. However, our case and control groups had similar diets, and therefore, differences in dietary lipids do not likely explain the differences observed. This suggests endogenous cause, i.e. altered absorption of dietary lipids or disturbed lipid metabolism, for the affected pathways in the fearful dogs.

Plasmalogens are important signalling molecules and free radical scavengers present in the majority of cell membranes [27, 44]. This family of ether-linked phospholipids has been heavily studied due to the potential anti-oxidant properties of plasmalogens [45, 46]. Previous studies of metabolic syndrome [47] and sepsis [48] patients have suggested decreased plasmalogen levels as a marker for oxidative stress. In the present study, fearful dogs had higher levels of PE(P-18:1/20:4) and it could be a secondary response for oxidative stress caused by chronic fear.

Besides plasmalogen, two other oxidative stress-related biomarkers were increased in fearful dogs: hypoxanthine and indoxylsulfate. Hypoxanthine is an oxidative stress stimulator [28, 29] and it effects are mediated by xanthine oxidase (XO), an enzyme which oxidases hypoxanthine to xanthine and further to uric acid. As a by-product of this process a highly deleterious superoxide is generated [30]. Indoxylsulfate promotes also oxidative stress [31, 32]. It is a uremic toxin metabolite of tryptophan that induces endothelial ROS production [32]. Oxidative stress is caused by an accumulation of reactive oxygen species (ROS), when the balance between pro- and antioxidant systems of the cell is disturbed [49]. As a result, several cellular components such as DNA, lipids, nucleic acids and proteins are damaged, and the levels of pro-inflammatory cytokines are increased. Oxidative stress has been associated with neuropsychiatric disorders like schizophrenia, anxiety, PTSD and social phobia across species [49–55]. There are also evidence that mitochondria-directed antioxidants relieve anxiety in rodents [56]. Further research is required to investigate the cause, whether primary or secondary, and significance of the elevated oxidative stress in the fearful dogs.

The third affected pathway was related to tryptophan metabolism. Fearful dogs had lower levels of tryptophan but increased levels of indoxylsulfate and hypaphorine. The latter two molecules are tryptophan metabolites. Hypaphorine (C_14_H_18_N_2_O_2_), an indole alkaloid and a betaine of tryptophan [33, 34] was greatly increased in fearful dogs. Biological functions of this metabolite are not well known and there is no link between hypaphorine and behavior. Since hypaphorine is a biomarker of consumption of pulses like beans and peas, increased hypaphorine could originate from diet. Unexpectedly, we found increase of hypaphorine in fearful dogs although dietary records indicated that control dogs had higher content of pulses in diet. This suggests that it is unlikely that such a significant and systematic difference in cases would result from nutrition solely. Instead, this observed change may refer to endogenic causes related to tryptophan metabolism, since hypaphorine is an N-methylated form of tryptophan. Also the identification of the other tryptophan metabolite indoxylsulfate supports the significance of altered tryptophan metabolism in fearful dogs. However, more research is needed to clarify the connection between these observed changes in canine anxiety.

This study demonstrates the promise of metabolomics approach in research related to canine anxiety, although we recognize technical and theoretical limitations that could be improved in future studies. First, we used whole blood and not plasma as a starting material. Whole blood challenges experimental conditions, including a sample preparation phase and may result in extra background followed by complications in downstream analyses. The replication study should be performed with fresh plasma samples collected in standardized manner. Second, the extraction conditions in the LC–MS platform were optimized for human samples and more optimal conditions should be investigated for samples of dog origin for higher quality of data. Third, better management of diet profiles of the participating dogs and sampling protocols should be considered in future experiments. The sampling time (morning/evening), the length of the sample storage time in the freezer and dog’s physical activity could have had effects on the metabolite profiles and should be controlled in future experiments. Finally, due to our small sample size but high amount of detected metabolic features, most of the observed changes were not significant after correction for multiplicity. Therefore, too far conclusions cannot be drawn from these results, and larger cohorts are needed although require more efforts for preparation given that we research private pets not colony dogs. However, despite the heterogeneous background and conditions of this pilot study, we were clearly able to identify several anxiety relevant metabolites in fearful Great Danes and thereafter warrant the future applications of metabolomics investigations.

## Conclusions

In summary, the pilot non-targeted metabolite profiling of canine anxieties indicates significant differences between fearful and non-fearful dogs. 13 identified metabolites were differential in the whole blood of fearful dogs, and are involved in oxidative stress, tryptophan and lipid metabolisms. Furthermore, these changes appear relevant to anxiety also in other species. This study demonstrates the power of the non-targeted metabolite profiling approach and encourages for a replication in a larger cohort of dogs with anxiety. Reliable replication of the identified biomarkers and pathways in this study could lead to applications for improved phenotyping and understanding of anxiety across species.

## Abbreviations

ESI: electrospray ionization
HILIC: hydrophilic interaction
LC: liquid chromatography
LysoPC: lysophosphatidylcholine
LysoPE: lysophosphatidylethanolamine
MS: mass spectrometry
PC: phosphatidylcholine
PLS-DA: partial least squares discriminant analysis
RP: reversed phase
VIP: variable influence on projection
